# Supplementary material for: Unveiling the Bioactive Potential of the Invasive Jellyfish Phyllorhiza punctata Through Integrative Transcriptomic and Proteomic Analyses
Source: Biomolecules. 2025 Aug 4;15(8):1121. doi: 10.3390/biom15081121 (PMC12383608; doi:10.3390/biom15081121)
Supplement: Supplementary file 1 [file biomolecules-15-01121-s001.zip › Suplementary Material/FileS3/PEPFOLD4/XP_065058271_7390/clusters.html]

 PEP-FOLD clusters


Download archive of all models  here   

|  |  |  |
| --- | --- | --- |
| # | file | sOPEP |
| # cluster 1 - Size: 2 |  |  |
|  | XP\_065058271\_7390-model1 | -29.0417 |
|  | XP\_065058271\_7390-model1.1 | -28.6784 |
| # cluster 2 - Size: 1 |  |  |
|  | XP\_065058271\_7390-model2 | -25.838 |
| # cluster 3 - Size: 8 |  |  |
|  | XP\_065058271\_7390-model3 | -25.67 |
|  | XP\_065058271\_7390-model3.1 | -25.1669 |
|  | XP\_065058271\_7390-model3.2 | -24.9199 |
|  | XP\_065058271\_7390-model3.3 | -24.7796 |
|  | XP\_065058271\_7390-model3.4 | -24.7526 |
|  | XP\_065058271\_7390-model3.5 | -24.7088 |
|  | XP\_065058271\_7390-model3.6 | -24.4099 |
|  | XP\_065058271\_7390-model3.7 | -23.6391 |
| # cluster 4 - Size: 23 |  |  |
|  | XP\_065058271\_7390-model4 | -25.542 |
|  | XP\_065058271\_7390-model4.1 | -25.2456 |
|  | XP\_065058271\_7390-model4.2 | -25.1461 |
|  | XP\_065058271\_7390-model4.3 | -25.104 |
|  | XP\_065058271\_7390-model4.4 | -24.957 |
|  | XP\_065058271\_7390-model4.5 | -24.937 |
|  | XP\_065058271\_7390-model4.6 | -24.9106 |
|  | XP\_065058271\_7390-model4.7 | -24.8732 |
|  | XP\_065058271\_7390-model4.8 | -24.8527 |
|  | XP\_065058271\_7390-model4.9 | -24.8219 |
|  | XP\_065058271\_7390-model4.10 | -24.8178 |
|  | XP\_065058271\_7390-model4.11 | -24.768 |
|  | XP\_065058271\_7390-model4.12 | -24.7565 |
|  | XP\_065058271\_7390-model4.13 | -24.7222 |
|  | XP\_065058271\_7390-model4.14 | -24.6708 |
|  | XP\_065058271\_7390-model4.15 | -24.6501 |
|  | XP\_065058271\_7390-model4.16 | -24.604 |
|  | XP\_065058271\_7390-model4.17 | -24.5929 |
|  | XP\_065058271\_7390-model4.18 | -24.5399 |
|  | XP\_065058271\_7390-model4.19 | -24.3831 |
|  | XP\_065058271\_7390-model4.20 | -24.3348 |
|  | XP\_065058271\_7390-model4.21 | -24.2382 |
|  | XP\_065058271\_7390-model4.22 | -24.0722 |
| # cluster 5 - Size: 2 |  |  |
|  | XP\_065058271\_7390-model5 | -25.4871 |
|  | XP\_065058271\_7390-model5.1 | -24.5776 |
| # cluster 6 - Size: 19 |  |  |
|  | XP\_065058271\_7390-model6 | -25.3836 |
|  | XP\_065058271\_7390-model6.1 | -25.2612 |
|  | XP\_065058271\_7390-model6.2 | -25.2532 |
|  | XP\_065058271\_7390-model6.3 | -25.1422 |
|  | XP\_065058271\_7390-model6.4 | -25.0792 |
|  | XP\_065058271\_7390-model6.5 | -24.9422 |
|  | XP\_065058271\_7390-model6.6 | -24.9124 |
|  | XP\_065058271\_7390-model6.7 | -24.7749 |
|  | XP\_065058271\_7390-model6.8 | -24.7443 |
|  | XP\_065058271\_7390-model6.9 | -24.7274 |
|  | XP\_065058271\_7390-model6.10 | -24.5793 |
|  | XP\_065058271\_7390-model6.11 | -24.5554 |
|  | XP\_065058271\_7390-model6.12 | -24.5248 |
|  | XP\_065058271\_7390-model6.13 | -24.5247 |
|  | XP\_065058271\_7390-model6.14 | -24.4762 |
|  | XP\_065058271\_7390-model6.15 | -24.4124 |
|  | XP\_065058271\_7390-model6.16 | -24.2873 |
|  | XP\_065058271\_7390-model6.17 | -24.2598 |
|  | XP\_065058271\_7390-model6.18 | -23.8049 |
| # cluster 7 - Size: 2 |  |  |
|  | XP\_065058271\_7390-model7 | -25.2161 |
|  | XP\_065058271\_7390-model7.1 | -24.9334 |
| # cluster 8 - Size: 10 |  |  |
|  | XP\_065058271\_7390-model8 | -25.0287 |
|  | XP\_065058271\_7390-model8.1 | -24.9425 |
|  | XP\_065058271\_7390-model8.2 | -24.8993 |
|  | XP\_065058271\_7390-model8.3 | -24.8365 |
|  | XP\_065058271\_7390-model8.4 | -24.7613 |
|  | XP\_065058271\_7390-model8.5 | -24.5941 |
|  | XP\_065058271\_7390-model8.6 | -24.5806 |
|  | XP\_065058271\_7390-model8.7 | -24.5193 |
|  | XP\_065058271\_7390-model8.8 | -24.5148 |
|  | XP\_065058271\_7390-model8.9 | -24.443 |
| # cluster 9 - Size: 1 |  |  |
|  | XP\_065058271\_7390-model9 | -24.9736 |
| # cluster 10 - Size: 3 |  |  |
|  | XP\_065058271\_7390-model10 | -24.9384 |
|  | XP\_065058271\_7390-model10.1 | -24.5963 |
|  | XP\_065058271\_7390-model10.2 | -24.4497 |
| # cluster 11 - Size: 3 |  |  |
|  | XP\_065058271\_7390-model11 | -24.5454 |
|  | XP\_065058271\_7390-model11.1 | -24.4204 |
|  | XP\_065058271\_7390-model11.2 | -24.0125 |
| # cluster 12 - Size: 1 |  |  |
|  | XP\_065058271\_7390-model12 | -23.4555 |
| # cluster 13 - Size: 1 |  |  |
|  | XP\_065058271\_7390-model13 | -22.348 |
| # cluster 14 - Size: 1 |  |  |
|  | XP\_065058271\_7390-model14 | -21.2082 |
| # cluster 15 - Size: 1 |  |  |
|  | XP\_065058271\_7390-model15 | -20.2715 |
| # cluster 16 - Size: 2 |  |  |
|  | XP\_065058271\_7390-model16 | -18.752 |
|  | XP\_065058271\_7390-model16.1 | -18.1379 |
| # cluster 17 - Size: 2 |  |  |
|  | XP\_065058271\_7390-model17 | -18.4798 |
|  | XP\_065058271\_7390-model17.1 | -18.0293 |
| # cluster 18 - Size: 1 |  |  |
|  | XP\_065058271\_7390-model18 | -17.6884 |
| # cluster 19 - Size: 1 |  |  |
|  | XP\_065058271\_7390-model19 | -17.3366 |
| # cluster 20 - Size: 2 |  |  |
|  | XP\_065058271\_7390-model20 | -17.0748 |
|  | XP\_065058271\_7390-model20.1 | -16.7732 |
| # cluster 21 - Size: 1 |  |  |
|  | XP\_065058271\_7390-model21 | -17.0325 |
| # cluster 22 - Size: 1 |  |  |
|  | XP\_065058271\_7390-model22 | -16.8501 |
| # cluster 23 - Size: 1 |  |  |
|  | XP\_065058271\_7390-model23 | -16.7508 |
| # cluster 24 - Size: 2 |  |  |
|  | XP\_065058271\_7390-model24 | -16.3455 |
|  | XP\_065058271\_7390-model24.1 | -15.2934 |
| # cluster 25 - Size: 1 |  |  |
|  | XP\_065058271\_7390-model25 | -16.1603 |
| # cluster 26 - Size: 4 |  |  |
|  | XP\_065058271\_7390-model26 | -15.9166 |
|  | XP\_065058271\_7390-model26.1 | -15.774 |
|  | XP\_065058271\_7390-model26.2 | -15.1986 |
|  | XP\_065058271\_7390-model26.3 | -15.0791 |
| # cluster 27 - Size: 1 |  |  |
|  | XP\_065058271\_7390-model27 | -15.6624 |
| # cluster 28 - Size: 1 |  |  |
|  | XP\_065058271\_7390-model28 | -14.8962 |
| # cluster 29 - Size: 1 |  |  |
|  | XP\_065058271\_7390-model29 | -13.5946 |
| # cluster 30 - Size: 1 |  |  |
|  | XP\_065058271\_7390-model30 | -12.1026 |
|  |  |  |
